# Metabolomic and Functional Genomic Analyses Reveal Varietal Differences in Bioactive Compounds of Cooked Rice

**DOI:** 10.1371/journal.pone.0012915

**Published:** 2010-09-23

**Authors:** Adam L. Heuberger, Matthew R. Lewis, Ming-Hsuan Chen, Mark A. Brick, Jan E. Leach, Elizabeth P. Ryan

**Affiliations:** 1 Department of Soil and Crop Sciences, Colorado State University, Fort Collins, Colorado, United States of America; 2 Proteomics and Metabolomics Facility, Department of the Vice President for Research, Colorado State University, Fort Collins, Colorado, United States of America; 3 Rice Research Unit, Agricultural Research Service, United States Department of Agriculture, Beaumont, Texas, United States of America; 4 Department of Bioagricultural Sciences and Pest Management, Colorado State University, Fort Collins, Colorado, United States of America; 5 Department of Clinical Sciences, Colorado State University, Fort Collins, Colorado, United States of America; University of Melbourne, Australia

## Abstract

Emerging evidence supports that cooked rice (*Oryza sativa* L.) contains metabolites with biomedical activities, yet little is known about the genetic diversity that is responsible for metabolite variation and differences in health traits. Metabolites from ten diverse varieties of cooked rice were detected using ultra performance liquid chromatography coupled to mass spectrometry. A total of 3,097 compounds were detected, of which 25% differed among the ten varieties. Multivariate analyses of the metabolite profiles showed that the chemical diversity among the varieties cluster according to their defined subspecies classifications: *indica, japonica*, and *aus*. Metabolite-specific genetic diversity in rice was investigated by analyzing a collection of single nucleotide polymorphisms (SNPs) in genes from biochemical pathways of nutritional importance. Two classes of bioactive compounds, phenolics and vitamin E, contained nonsynonymous SNPs and SNPs in the 5′ and 3′ untranslated regions for genes in their biosynthesis pathways. Total phenolics and tocopherol concentrations were determined to examine the effect of the genetic diversity among the ten varieties. Per gram of cooked rice, total phenolics ranged from 113.7 to 392.6 µg (gallic acid equivalents), and total tocopherols ranged between 7.2 and 20.9 µg. The variation in the cooked rice metabolome and quantities of bioactive components supports that the SNP-based genetic diversity influenced nutritional components in rice, and that this approach may guide rice improvement strategies for plant and human health.

## Introduction

Rice (*Oryza sativa* L.) is a valuable model system for cereal plant genetics due to its sequenced and annotated genome, capacity for transformation, and similarity to other major cereal crop species. Most importantly, rice is a vital source of calories as a food crop. Cereals are the primary source of energy for over 50% of the global population, of which rice is the third largest contributor [1]. The global dependence on rice has led to the development of thousands of varieties with large genetic and morphological diversity. Rice is structured into several well-defined gene pools via the subspecies classification of *indica, japonica*, and *aus*. This classification was recently confirmed with the genome resequencing of 20 representative varieties and subsequent documentation of single nucleotide polymorphisms (SNPs), referred to as the OryzaSNP set [2]. Across and within each classification, rice contains significant diversity in plant architecture and growing habits [3], and in grain phenotypes such as width, weight, cooking properties, aroma, and texture [4]. The extensive phenotypic and genotypic variation within the OryzaSNP set makes these varieties a powerful tool to study rice chemical diversity such that methods can be developed to enhance health promoting qualities of rice.

Metabolites present in the rice grain have demonstrated human disease protective activities following dietary intake, and also have beneficial effects on the immune system [5]–[7]. Specific rice components, such as phenolics (mono- and polyphenols), vitamin E (tocopherols and tocotrienols), phytosterols, and linolenic acid, have nutrient value to human health [8]–[11]. Phenolic bioactivity is largely due to the efficiency of donating hydrogen atoms to oxygen radicals [12], a process associated with anticancer activity [13]. Unlike phenolics, tocopherols are lipid-soluble antioxidants incorporated into lipoproteins, and are predicted to counteract the inflammatory effects of lipoprotein oxidation in blood [14]. While brown rice is an efficient source of both phenolics and tocopherols, little is known regarding the genetic basis for the variation in type and quantity of these components in cooked rice across genetically diverse varieties.

The functional impact of SNP-derived genetic variation in pathways that regulate the production of dietary bioactive compounds in rice is also unclear. Metabolomics, the comprehensive analysis of low-molecular-weight compounds in biological samples, provides a high-throughput and sensitive approach to assess the outcome of different genotypes on metabolites in the cooked grain. New evidence supports the utility of this technique to capture the complexity of the rice metabolome and to evaluate changes in metabolic responses [15], [16]. However, there has been minimal integration of the rice metabolomic signature with genomic data sets and the use of this information to assess components of dietary importance. A systems biology approach was applied herein to reveal the synthesis and metabolic regulation of nutritionally important phytochemicals, by profiling multiple rice varieties for pathway-specific SNPs with metabolomics.

## Results

### Metabolite variation in rice varieties and subspecies

**Figure 1:**
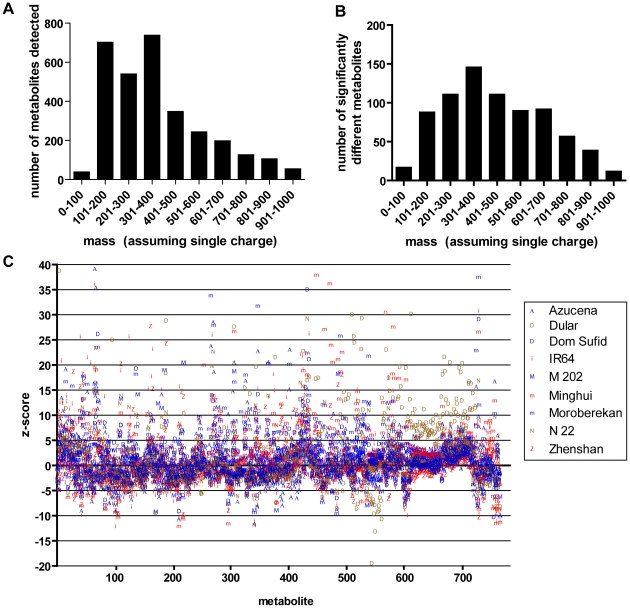
Metabolite detection across ten rice varieties. (A) Rice metabolites were detected by UPLC-MS and all 3,097 metabolites were sorted by size. (B) The 763 metabolites that differ among the ten varieties were dispersed across a similar mass distribution as the total metabolite profile. (C) Z-score analysis on the 763 metabolites was conducted using Nipponbare (*japonica*) as a reference. *Indica*, *japonica*, and *aus* varieties are shown in red, blue, and brown, respectively. A total of 32 data points with a z-score of greater than 40 were outside of the area shown.

**Figure 2:**
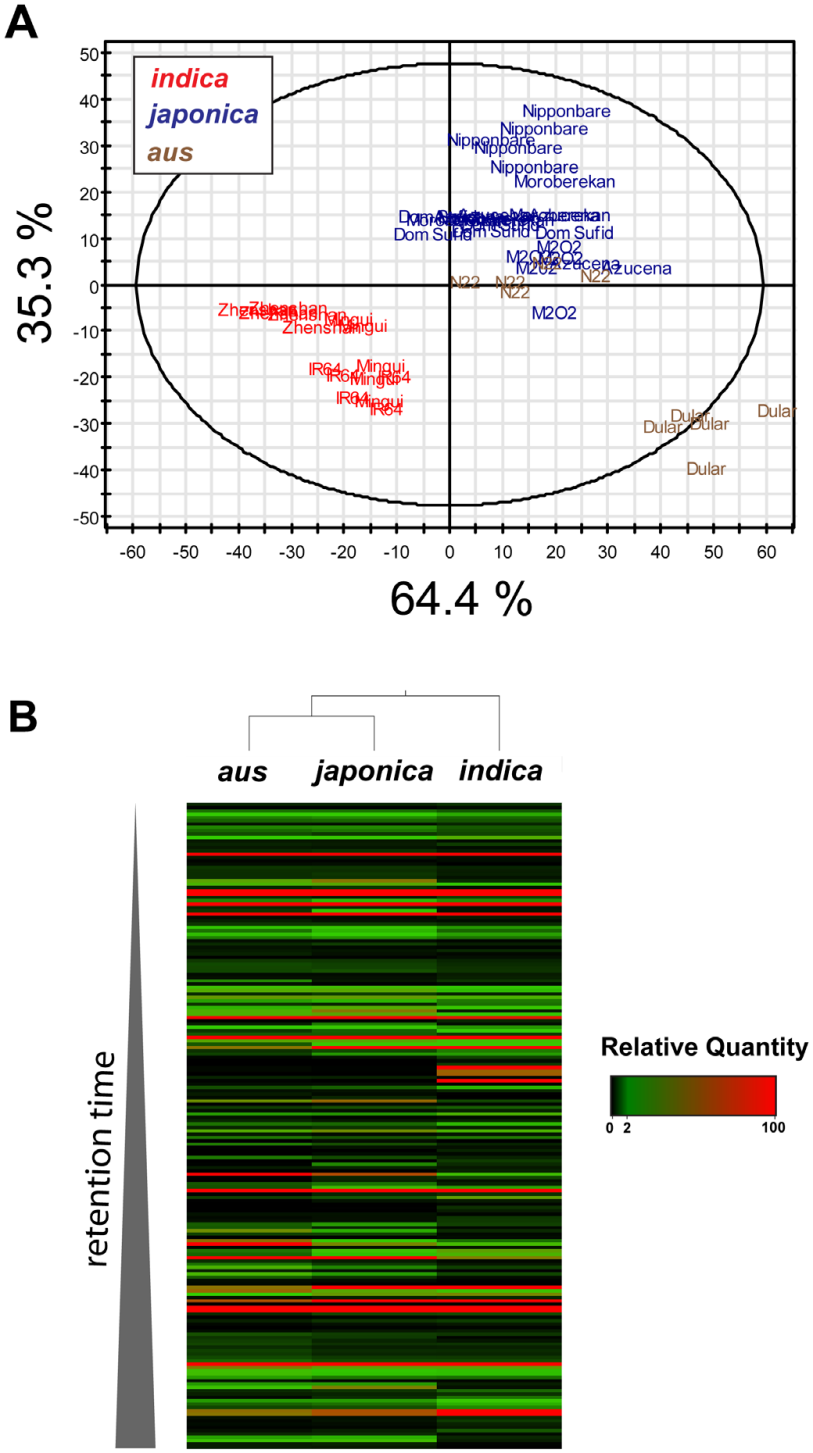
Subspecies analysis of the cooked rice metabolome. (A) PLS-discriminant analysis was conducted on ten rice varieties and was colored according to subspecies as *indica* (red), *japonica* (blue), and *aus* (brown). (B) The 194 metabolites that differ among the three subspecies were shown in a heat map whereby each cell represents a single metabolite. Metabolites were arranged according to retention time (0.5–12 minutes), and colors indicate relative quantities. Hierarchical clustering was performed using Euclidean distances.

A comparison of metabolite profiles was conducted to determine the extent of variation in cooked brown rice across ten varieties from the OryzaSNP set (Table 1). The subset of OryzaSNP varieties used in this study represent the extensive phenotypic and genetic diversity present in the three subspecies (*aus, japonica, indica*) of consumed rice varieties [2]. They also represent different levels of improvement through breeding [3]. Metabolites from cooked brown rice were extracted in 80∶20 methanol:water and detected by ultra performance liquid chromatography coupled with mass spectrometry (UPLC-MS). A metabolomic profile for each rice variety was resolved as a sum of its features, and each feature (assumed here to be a unique metabolite) consists of a retention time, mass, and quantity. Across the ten varieties, 3,097 metabolites were detected, and these metabolites were distributed across a wide range of molecular masses (Figure 1A). Approximately 25% (763 out of 3,097) of the metabolites differed in quantity among the ten varieties (Kruskal-Wallis test, *P*<0.001) (Figure 1B). A z-score analysis applied to the set of 763 metabolites showed extensive metabolite variation relative to Nipponbare, a Japanese variety with a sequenced genome (Figure 1C). A sum of squares for the 763 z-scores showed that the metabolite profiles of all nine varieties were different from the profile of Nipponbare, and that profiles of *indica* subspecies varieties show larger differences from Nipponbare than did *japonica* subspecies profiles (Table 2). Based on a partial least squares discriminant analysis (PLS-DA), metabolite profiles cluster according to subspecies (*indica, japonica, aus*) (Figure 2A). The first component of the PLS-DA model explained approximately 64% of the variation, and the second component explained an additional 35% of the variation. Varieties were then clustered into the *indica, japonica*, and *aus* subspecies, and 194 metabolites were determined to be significantly different among the three subspecies (Kruskal-Wallis test, *P*<0.001) (Figure 2B). Hierarchical clusters were determined using Euclidian distances, and the metabolite profiles of the *aus* varieties were nearer to the *japonica* than the *indica* varieties. The differences in the chemical profiles among the ten varieties suggest the potential for variation in metabolites important for human nutrition.

**Table 1:** Rice materials.

Variety	Country of Origin	Subspecies	IRGC Accession ID	Breeding Classification	Traits of interest
Azucena	Philippines	*japonica*	117264	Landrace	Fragrant, tall stature, unique root structure
Dular	India	*aus*	117266	Landrace	Drought resistant, seed-shattering
Dom-Sufid	Iran	*japonica*	117265	Landrace	Similar to Basmati rice (aromatic)
IR64-21	Philippines	*indica*	117268	Advanced	Widely grown, semidwarf, high yielding, abiotic and biotic stress tolerance
M 202	United States	*japonica*	117270	Advanced	Erect leaf type, modern variety
Minghui 63	China	*indica*	117271	Advanced	Parent used in hybrid breeding
Moroberekan	Guinea	*japonica*	117272	Landrace	Abiotic and biotic stress tolerance
N22	India	*aus*	117273	Landrace	Red seed coat, stress tolerance
Nipponbare	Japan	*japonica*	117274	Advanced	First sequenced variety, short grain type
Zhenshan 97B	China	*indica*	117280	Advanced	Parent used in hybrid breeding

**Table 2:** Sum of squares of z-scores using Nipponbare (*japonica*) as a reference.

Variety	Class	Sum of Squares
Zhenshan	*indica*	49,099,871
Minghui	*indica*	6,683,571
IR64	*indica*	4,709,880
Dom Sufid	*japonica*	568,329
Azucena	*japonica*	273,295
M 202	*japonica*	79,634
Moroberekan	*japonica*	31,872
Dular	*aus*	71,600
N22	*aus*	19,577

### SNP analysis reveals allelic differences in phytochemical pathways of nutritional importance in rice

Relevant metabolic pathways, including those involved in the biosynthesis of phenolics, vitamin E, phytosterols, and linolenic acid, were chosen for functional genomic analysis of SNPs across the diverse rice varieties. The RiceCyc database (www.pathway.gramene.org/rice) was used to align the four classes of metabolites to biochemical pathways, and then to identify genes from the associated chemical reactions (Table S1). Pathways for phenolics combined both phenylpropanoid and flavanoid synthesis due to conservation of structure and function, and also included isoflavone-7-O-methytransferase 9 and leucodelphinidin biosynthesis genes that synthesize tricin, a phenolic unique to rice [17]. Vitamin E genes encode components of the tocopherol and tocotrienol synthesis pathways, which includes α-, β-, γ-, δ-tocopherol and tocotrienol-related enzymes, as well as tocopherol O-methyltransferase and homogentisic acid geranylgeranyl transferase genes [18]. Genes involved in phytosterol synthesis were derived from sterol synthesis pathways [19], and linoleic acid genes were derived from lipid desaturation pathways [20]. Genes were screened for SNPs using the rice OryzaSNP database (www.oryzasnp.org), which classified rice SNPs based on up to four gene models.

SNPs, base calls, and SNP classifications were associated to their respective class of metabolites by cross-referencing locus identifiers to the metabolite pathway database. A number of SNPs were detected in pathways associated with synthesis of metabolites important to human health (Table 3). SNPs in gene pathways responsible for the synthesis of phenolics were evenly distributed among synonymous, nonsynonymous, and intron classes. Phenolics also contained a greater amount of nonsynonymous SNPs per gene than phytosterols, vitamin E, or linolenic acid, and had a higher probability of a change in enzymatic function or regulation. One large-effect SNP is predicted to alter the function of the ferulate 5-hydroxylase enzyme (gene: LOC_Os06g24180) and was classified as potentially altering regular intron splicing events. A larger percentage of SNPs in the sterol, vitamin E, and linoleic acid pathways were within introns compared to phenolics.

**Table 3:** SNPs in genes that regulate phenolics, phytosterols, vitamin E, and linoleic acid.

Class	Genes	SNPs	SYN	NS	5′	3′	INT	SNPs/gene	NS SNPs/gene
Phenolics	30	78	24	22	2	12	17	2.60	0.73
Phytosterols	15	22	4	2	0	2	14	1.27	0.09
Vitamin E	9	23	2	2–4	3	2–3	14	2.55	0.09–0.17
Linolenate	7	8	0	0	0	1	7	1.14	0.00

SYN: synonymous.

NS: nonsynonymous.

5′, 3′: untranslated regions.

INT: intron.

**Figure 3:**
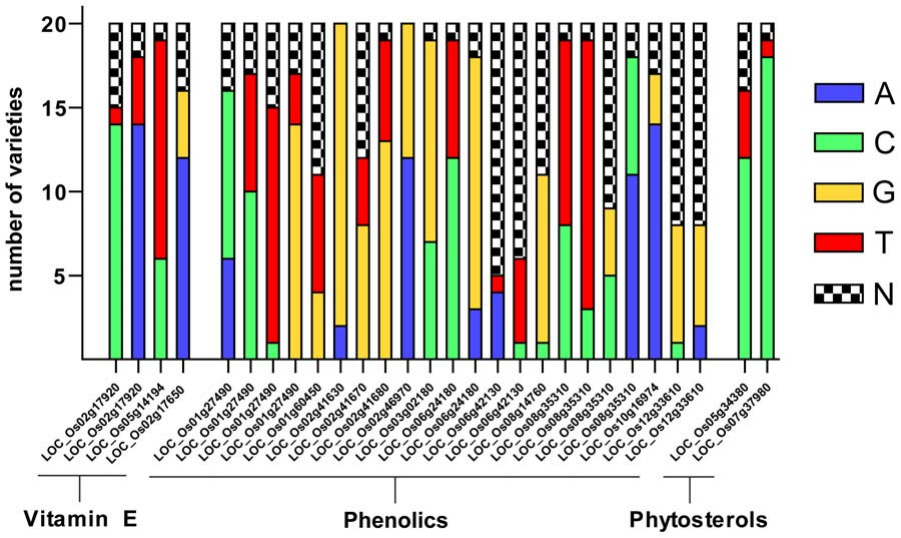
Allele frequencies within the OryzaSNP set for nonsynonymous SNPs in vitamin E, phenolic, and phytosterol pathways. Allele frequencies are represented as the number of SNPs in common for each of the 20 varieties of the OryzaSNP set. X-axis labels correspond to the rice locus identifier for a given SNP.

To identify the unique nonsynonymous SNPs in our rice collection, allele frequencies were calculated for genes involved in the phenolics and vitamin E biochemical pathways (Figure 3). Only two alleles existed for each of the 28 SNPs. Seven SNPs (25%) had one variety that contained its own unique allele. The remaining 21 SNPs (75%) had alleles that were shared among multiple varieties, and the average allele frequency per SNP was 0.52. The subset of 21 SNPs represent rice metabolic pathways that are common to a cluster of varieties rather than solitary occurrences.

### SNP diversity predicted subspecies variation in phenolics and vitamin E content

**Figure 4:**
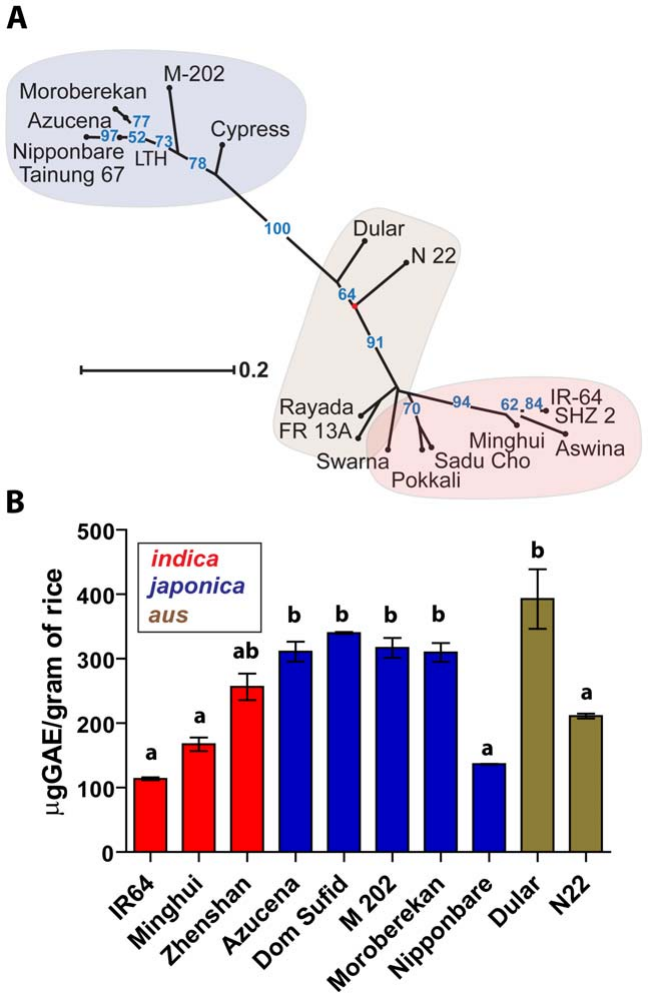
Variation in total phenolics concentrations in cooked rice. (A) An unrooted, neighbor-joining tree was developed based on total SNPs identified in the phenolic biochemical pathways. Clouds were colored according to subspecies: *indica* (red), *japonica* (blue), and *aus* (brown). (B) Total phenolics was measured in gallic acid equivalents (GAE) using Folin-Ciocalteau reagent. The letters a, b, and c denote significance (ANOVA, Tukey post-hoc, *P*<0.05), and values are expressed as the mean ± the standard error of the mean.

To further characterize the genetic control of nutritionally important metabolites, a dissimilarity matrix was constructed using a concatenated sequence of SNPs specific to phenolics or vitamin E pathways. The phytosterol and linolenic acid pathways had low SNP abundance, and therefore low variation (data not shown). Clustering based on SNPs in both phenolic and vitamin E pathways grouped the rice varieties according to the *indica, japonica*, and *aus* subspecies classifications (Figure 4A, 5A). The total phenolic concentration differed among the ten varieties (Figure 4B). The overall mean total phenolic concentration was 256 µg of gallic acid equivalents (GAE) g^−1^ of cooked rice. The variety Dular had the highest total phenolics with a mean of 393 µg GAE g^−1^ cooked rice. IR64 and Nipponbare had the least amounts with means of 114 and 136 µg GAE, respectively. The mean total phenolics was 179 µg GAE for the *indica*s, 288 µg GAE for the *japonicas*, and 302 µg GAE for the *aus* groups.

**Figure 5:**
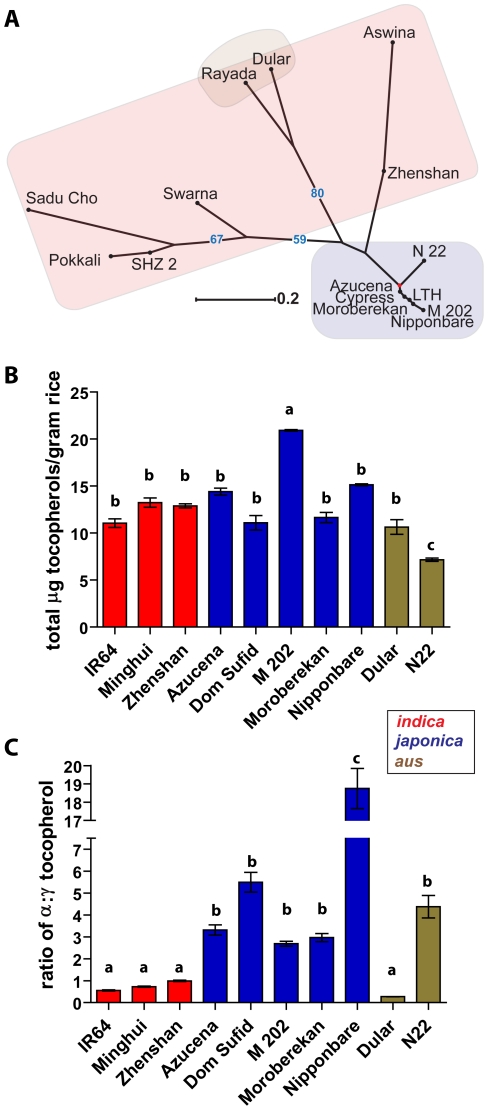
Variation in vitamin E concentrations in cooked rice. (A) An unrooted, neighbor-joining tree was developed based on total SNPs identified in the vitamin E synthesis pathway. Clouds were colored according to subspecies: *indica* (red), *japonica* (blue), and *aus* (brown). (B) The total quantities of tocopherols (α,γ, and δ) per gram of rice were determined. (C) Ratios of α:γ tocopherol were calculated for each variety. Values are expressed as the mean ± the standard error of the mean, and statistical groupings denoted by the letters a, b, and c (ANOVA, Tukey post-hoc, *P*<0.05).

The SNP diversity in vitamin E-relevant genes was larger for *indicas* than *japonicas* (Figure 5A). For vitamin E, the low mean number of nonsynonymous SNPs per gene predicted high conservation in total rice vitamin E concentration. The ten varieties of were analyzed for total tocopherols in the cooked grain, as well as the contribution by each of the main constituents: α-, γ-, and δ- tocopherol. N22 had the lowest levels of total tocopherols at 7.2 µg g^−1^ of cooked rice, and M202 had the highest concentration at 20.9 µg g^−1^ (Figure 5B). Because α-and γ-tocopherols vary in bioactivity, the contribution of α- and γ- to the total tocopherol pool was determined as a ratio of α:γ for each variety (Figure 5C). The levels of δ-tocopherol were consistently low and had a negligible contribution to total vitamin E. The ratio of α:γ significantly differed among the ten varieties. The *indica* varieties contained the highest levels of γ-tocopherols with a mean α:γ ratio of 0.75, whereas the *japonica* varieties contained higher levels of α-tocopherols with a mean ratio of 6.6. The variety Dular had the smallest α:γ ratio with a value of 0.27, and the Nipponbare variety had the largest α:γ ratio of 18.8. The tocopherol ratios of the two *aus* varieties (Dular and N22) were very different. None of the SNPs collected in Table S1 could directly explain the variation in tocopherol components. SNP diversity was smaller for predicting levels of vitamin E when compared to phenolics, however there was clear variation in the quantity of phenolics, and both the type and quantity of vitamin E metabolites among the ten rice varieties.

## Discussion

The diversity in genetic and morphological rice traits from the OryzaSNP set was interrogated herein by applying metabolomic analysis to the cooked grain. Previous studies have established metabolite profiles for crop varieties [21], [22], however metabolites were extracted from raw plant material. The screening of metabolites in cooked rice enhanced the dietary relevance of our findings, as the nutritional differences detected resembles actual metabolite intake following heat and moisture. An open-boiling technique was standardized for this study because of the global utilization of this cooking method.

Recent reviews emphasize the need for sustainable, breeding-based approaches to enhance plant food nutritional quality [23], [24]. An integrated genomic and metabolomic method has been proposed as a useful measure to improve food crops [25]. A number of studies successfully correlated genomics with metabolomics, such as in the associations of quantitative trait loci with metabolite profiles in *Arabidopsis* [26] and of restriction fragment length polymorphism markers with nuclear magnetic resonance-generated metabolite profiles in uncooked rice [22]. An analysis of SNPs provides a new functional relevance for the differences detected in the rice metabolome. The integration of SNP-based bioinformatics with metabolomics as conducted herein may now be utilized to assist in selection of rice varieties with enhanced nutritional and health-promoting value.

The extensive metabolite variation in different varieties of cooked rice was approximately 25% of the total metabolites detected. The z-score analysis using Nipponbare as a reference was a compelling example of the metabolite diversity among the varieties (Figure 1C). Z-scores were calculated to determine metabolites that vary between one variety and a reference variety. An excessively high or low z-score (roughly higher or lower than five) usually indicated a metabolite present in one variety and absent in another, and may provide direction in identifying unique metabolites. The sum of squares of the z-scores suggested that the *indica* varieties were more different from Nipponbare than the *japonica* or *aus*, and was expected given that Nipponbare is a *japonica* variety.

Another strong link between the rice genome diversity and cooked rice metabolome was the PLS-DA model that clustered the cooked rice metabolome for each variety according to subspecies (Figure 2A). Genomewide, *aus* is more homologous to the *indica* subspecies [2], however N22 (*aus*) grouped closely with the *japonicas* following metabolite analysis with both z-scores and the PLS-DA. The hierarchical clustering of the 763 metabolites that represent total metabolite variation also grouped the *aus* varieties closer to the *japonicas* than the *indicas*. This contrast between observed genomic homology and metabolomic profiles is likely due to introgressions of metabolite-related loci into the *aus* background. Such introgressions are frequent in rice [27], and have been utilized for genetic association strategies to identify loci important for synthesizing trait-specific metabolites in *Arabidopsis* [28] and tomato [29].

The genes in nutritionally important biochemical pathways contained SNP variation (Figure 4A, 5A) that correlated with the UPLC-MS-derived metabolome for cooked rice (Figure 2A). SNPs with functionally-relevant classifications were found in genes in the phenolics, vitamin E, phytosterol, and linolenic acid pathways, with a larger mean number of SNPs per gene in the phenolics and vitamin E pathways. The total number of nonsynonymous SNPs may be larger than described in Table 3 because many genes and enzymes for key biochemical reactions remain unknown. Furthermore, our SNP analysis was limited to a subset of rice varieties that were diverse but represent a small proportion of the total rice genetic diversity.

The SNP homology in the phenolic and vitamin E pathways for the ten rice varieties coincided with the *indica, japonica*, and *aus* subspecies classifications. However, the phytosterol and linolenic acid pathways lacked sufficient information to function in a SNP homology-based model. The SNP dendrograms for phenolic and vitamin E related metabolites predicted that a given rice variety will be more similar to a variety of the same subspecies than of another subspecies. It can be postulated that distinct haplotypes for the synthesis and regulation of nutritionally important phytochemicals are present in select rice varieties. Thus, it is plausible that a SNP haplotype was responsible for a given variety's metabolite profile, and that haplotype breeding approaches could be used to optimize the metabolite profiles of rice for nutritionally important health traits.

The total phenolic concentration varied both among and within subspecies. In general, the *japonica* varieties contained a higher level of total phenolics (288 µg GAE g^−1^) than the *indicas* (179 µg GAE g^−1^). However, Nipponbare contained a lower abundance of phenolics (136 µg GAE g^−1^) than its *japonica* counterparts, and Zhenshan appears an *indica*-outlier due to its higher concentration of total phenolics (256 µg GAE g^−1^) than other *indicas*. This was consistent with the z-score analysis, in which Zhenshan contained the largest difference from Nipponbare (Table 2). N22 (*aus*) also grouped with the *indicas*, and both the z-score and PLS-DA models grouped N22 closer to Nipponbare (*japonica*) than Dular (*aus*). It is possible that a proportion of the variation in the z-score and PLS-DA models was due to differences in phenolics, as the solvents used herein have been shown to extract phenolic compounds from rice [30], [31].

The total quantity of tocopherols per gram of rice showed slight variation among the ten varieties, and the observed range in quantities were similar to those found in various plants and plant tissues [32]. The α and γ forms of tocopherol have different bioactive functions and metabolism [33]–[35]. The tendency for the α:γ ratio to link a variety within its subspecies is consistent with the observed trends in both the metabolome (Figure 2A) and total phenolics (Figure 4B) analyses, however specific varieties also deviate from the larger subspecies trends (Figure 5C). For the α:γ ratio, N22 (*aus*) clusters closer to all *japonicas* except for Nipponbare (*japonica*). Dular (*aus*) clusters with the *indicas*, which all contain a lower ratio of α:γ tocopherol than the *japonicas*. SNPs were not able to explain the *indica*/*japonica* division in α:γ ratios, as none were identified in γ-tocopherol-O-methyltransferase (γ-TMT), the enzyme that converts γ- to α-tocopherol by the addition of a methyl group. Enhanced γ-TMT expression has been shown to increase the α:γ ratios in various plants and tissues, but does not alter the overall quantity of tocopherols [36]–[38]. Thus, the variation among the ten rice varieties may be due to differential γ-TMT gene expression rather than a SNP-driven change in function. Furthermore, the α:γ tocopherol ratios were consistent with observed ratios of tocotrienols (data not shown), which further supports the importance of the γ-TMT in determining the overall composition of vitamin E. SNPs were not identified in the 5′ untranslated region of the rice γ-TMT gene, and therefore it is likely that a diverse set of vitamin E gene regulators exists for tocopherol accumulation in rice.

The identification of the genetic basis for important agronomic traits, such as yield and abiotic/biotic stresses has led to considerable advances in accumulating desirable traits into rice breeding programs. The incorporation of nutritional traits, however, has been principally overlooked due to an emphasis on total plant yield [39]. Here, the findings provide evidence for regular, systematic evolution at loci important to nutritional metabolite synthesis. A deeper understanding of the genetic basis for the type and quantity of metabolites in the rice grain may allow for breeding plants that contain an optimal metabolite profile for enhanced health attributes.

## Materials and Methods

### Rice materials

Rice seeds were acquired from the International Rice Research Institute (IRRI, Los Baños, Philippines) and are listed in Table 1. Rice plants were grown at the Dale Bumpers National Rice Research Center in Stuttgart, Arkansas to produce seed used in this study. The grain was isolated from the husk using a manual stone dehusker and then cooked by boiling in a 2∶1 volume of water/rice ratio for 15 minutes or until soft. Cooked rice was lyophilized over a period of 48 hours immediately after cooking and stored at −80°C until further analysis.

### Rice processing and extractions

Metabolites in cooked rice were extracted by first grinding rice to a powder with a mortar and pestle in liquid nitrogen. One mL of ice-cold methanol/water (4∶1) was added to 100 mg of rice powder. Samples were incubated for one hour at −80°C to precipitate protein, centrifuged at 1500×g for five minutes at 4°C, and the supernatant was collected and stored at −20°C until further analysis.

### Ultra Performance Liquid Chromatography-Mass spectrometry

Rice extract separation was performed using an Acquity UPLC® controlled with MassLynx software, version 4.1 (Waters, Milford, MA, USA). Samples were held at 8°C in a sample manager during the analysis to minimize evaporation. The complete sample set was randomized and profiled in two independent iterations. Sample injections of 2 µL were made to a 1.0×100 mm Waters Acquity UPLC® BEH C8 column with 1.7 µm particle size held at 40°C. Separation was performed by reverse phase chromatography at a flow rate of 0.14 mL/min. The eluent consisted of water and methanol (Fisher, Optima LC-MS grade) supplemented with formic acid (Fluka, LC-MS grade) in the following proportions: Solvent A = 95∶5 water:methanol +0.1% formic acid; Solvent B = 5∶95 water:methanol +0.1% formic acid. The separation method is described as follows (25 minutes total): 0.1 min hold at 30% B, 1.9 min linear gradient to 70% B, ten min linear gradient to 100% B, 6 min hold at 100% B, 0.1 min linear gradient to 30% B, and 6.9 min hold at 30% B for column equilibration prior to the next injection.

Eluate was directed to a Q-TOF Micro quadrupole orthogonal acceleration time-of-flight mass spectrometer (Waters/MicroMass, Millford, MA, USA) using positive mode electrospray ionization (ESI+). Mass data were collected between 50 and 1000 m/z at a rate of one scan per second. The voltage and temperature parameters were tuned for general profiling as follows: capillary  = 3000 V; sample cone  = 30 V; extraction cone  = 2.0 V; desolvation temperature  = 300°C; and source temperature  = 130°C. Mass spectral scans were centered in real time producing centroid data. Leucine Enkephalin was infused via a separate orthogonal ESI spray and baffle system (LockMass) which allowed ions to be detected for a single-second scan every ten seconds in an independent data collection channel. The standard mass was averaged across ten scans providing a continuous reference for mass correction of analyte data.

### Allele frequencies

Allele frequencies were calculated for each SNP site based on the 20 varieties of the OryzaSNP set (www.oryzasnp.org). Base calls for each SNP were determined using TIGR Pseudomolecule v5 in the OryzaSNP database. Frequencies were determined by evaluating the proportion of adenine, guanine, cytosine, and thymine nucleotides among the 20 varieties for each SNP site. Unresolved nucleotides were reported as “N.” Genes in the vitamin E pathway contained between two, three, or four nonsynonymous SNPs based on different gene models, and all gene models were analyzed for allele frequency calculations.

### SNP Dendrogram

An unweighted, unrooted neighbor-joining tree with 1000 bootstraps was constructed using DARwin (http://darwin.cirad.fr/darwin). Inputs for each variety consisted of a collection of base calls specific to either the phenolic or vitamin E pathway. For each pathway, SNP sites with greater than 50% unknown nucleotides were not included in the analysis, and varieties with greater than 50% missing information were also removed.

### Total Phenolics Assay

Total phenolic concentrations in rice extracts were determined as previously described [40] with minor modifications. Briefly, 150 µL of Folin-Ciocalteu reagent/water (1∶9) was added to 35 µL of rice metabolite extract and was incubated at room temperature for five minutes. Sodium bicarbonate (115 µL of a 7.5% solution) was then added and samples were incubated at 37°C for 30 minutes. Samples were allowed to cool to room temperature and absorbance was measured at 765 nm. Metabolite extractions were performed in triplicate. Total phenolics were calculated using a standard curve genereated using a series of gallic acid concentrations and were expressed as micrograms of gallic acid equivalents (GAE) per gram of rice.

### Vitamin E quantification

Tocopherol homologs, α-, γ-, and δ-tocopherols, were purchased from Cayman Chemicals (Ann Arbor, MI; ≥98% purity). Tocotrienol homologs, α-, γ-, and δ-tocotrienols, were purchased from Matreya Biochemicals (Pleasant Gap, PA; ≥97% purity). Methanol and acetonitrile were HPLC grade from Fisher Scientific (Fair Lawn, NJ).

Tocopherols (α-,γ-, and δ-tocopherols) and tocotrienols (α-,γ-, and δ-tocotrienols) were determined using HPLC (Waters, Milford, MA) based on the method described in [41] with modifications. The HPLC was equipped with a Waters 2695 Alliance Separation Module, a Waters 2996 Photodiode array detector (PDA), a Waters 474 Scanning Fluorescence detector, and Empower™ 2 software for data acquisition. The cooked and lyophilized rice powders were extracted with 100% methanol twice at the bran to solvent ratio of 1 to 33 (w/v). For each extraction, the mixture was flushed with nitrogen gas and shaken (300 rpm) for 2 h at room temperature. After centrifugation at 2000×g for ten minutes at room temperature, the supernatants were pooled and filtered through a 0.45 µm polyvinylidene fluoride (PVDF) membrane (Waters, Milford, MA), injected through a Symmetryshield RP C-18 guard column (3.5 µm, 3.0×20 mm; Waters) and separated on a Symmetryshield RP C-18 analytical column (3.5 µm, 3.0×150 mm; Waters). The filtrate was eluted with a gradient mobile phase consisting of (A) 100% acetonitrile, (B) 100% methanol, and (C) 1% acetic acid in 50% methanol at 0.5 mL/min at 25°C. The gradient was used as follows: 0–1 min, 45% A, 35% B, and 20% C; 1–2 min, linear gradient to 45% A, 45% B, and 10% C; 2–16 min, linear gradient to 30% A, 65% B, and 5% C; 16–20 min, linear gradient to 25% A and 75% B; 20–22 min, linear gradient to 100% B; 22–25.4 min, isocratic at 100% B; 25.4–25.5 min, linear return to 45% A, 35% B, and 20% C; 25.5–35 min, isocratic at 45% A, 35% B, and 20% C to re-equilibrate. The tocopherol and tocotrienol homologs were detected by the fluorescence detector at the excitation and emission wavelengths of 298 and 328 nm, respectively. The peak identification of tocopherols and tocotrienols was performed by comparing their retention time with those of standards. The concentration of each tocopherol and tocotrienol homolog was calculated using the standard curve plotted as peak area against a series of concentrations of each tocopherol and tocotrienol homolog and indicated as µg/g rice. The coefficient of determinations (R^2^) ranged from 0.9962 to 0.9999. The β- and γ-forms of tocopherols and tocotrienols are isomers and co-elutes on reversed-phase C18 columns. Rice bran contains only trace amounts of β-form, nevertheless, the concentrations of γ-forms of tocopherols and tocotrienols in bran reflect the sum of β- and γ-forms in this study.

### Statistical Analysis

Chromatographic and spectral UPLC-MS peaks were detected, extracted, and aligned using MarkerLynx software (Waters, Millford, MA, USA). Chromatographic peaks were detected between 0 and 14 min with a retention time error window of 0.1 min. Apex track peak detection parameters were used, automatically detecting peak width and baseline noise. No smoothing was applied. To reduce the detection and inclusion of noise as data, an intensity threshold value of 40 counts and a noise elimination value of 6 were used. Mass spectral peaks were detected between 50 and 1000 m/z with a mass error window of 0.07 m/z, and the de-isotoping function was enabled. A matrix of features as defined by retention time and mass was generated, and the relative intensity (proportional to quantity) of each feature (metabolite), as determined by area of the peak, was calculated across all samples. Potential effects of instrument variability were minimized by normalizing the total ion current (TIC) among all samples such that the summation of all feature intensities in each sample yielded a constant value. Furthermore, the relative intensity of each feature was averaged over the two replicate injections preformed for each sample to provide a reliable data matrix with minimal technical artifacts. Mean centering was applied, and the data matrix was analyzed in SIMCA-P+ v. 11.5 (Umetrics, Umeå, Sweden). Pareto scaling was applied to the data, and a score plot was generated to describe the data using partial least squares discriminant analysis (PLS-DA). The PLS-DA model was validated by testing new PLS-DA models built from 20 random permutations of the data (Figure S1). Significant UPLC-MS-detected metabolites were determined using a Kruskal-Wallis test on relative intensities of features with identical masses and retentions times with a threshold value of *P*<0.001 and n = 5 replicates per variety. Z-scores were calculated for each metabolite based on the mean and standard deviation of the reference variety Nipponbare. Statistical significance for total phenolics and vitamin E analyses was determined by ANOVA with a Tukey post-test and a threshold value of *P*<0.05.

## Supporting Information

Figure S1 Validation of the partial least squares-discriminant analysis. The PLS-DA model for subspecies was validated using 20 permutations. Values for R^2^ (0.7) and Q^2^ (0.55) denote original and predictive data, respectively. A positive value of Q^2^ when R^2^ is zero (x-axis  = 0) would suggest overfit in the model.
(0.12 MB TIF)

Table S1 Genes associated with linolenic acid, phenolics, phytosterol, and vitamin E synthesis.
(0.18 MB PDF)
