# Supplementary figures and images for: Metabolomic and Functional Genomic Analyses Reveal Varietal Differences in Bioactive Compounds of Cooked Rice
Source: PLoS One. 2010 Sep 23;5(9):e12915. doi: 10.1371/journal.pone.0012915 (PMC2944836; doi:10.1371/journal.pone.0012915)

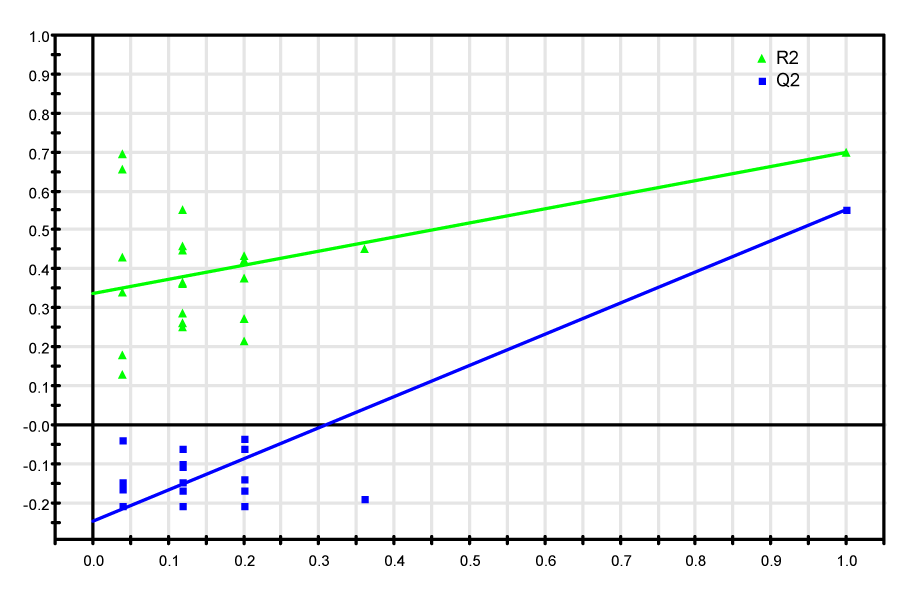

Supplement: Figure S1 — Validation of the partial least squares-discriminant analysis. The PLS-DA model for subspecies was validated using 20 permutations. Values for R2 (0.7) and Q2 (0.55) denote original and predictive data, respectively. A positive value of Q2 when R2 is zero (x-axis = 0) would suggest overfit in the model. (0.12 MB TIF) [file pone.0012915.s001.tif]
