# Supplementary material for: Metabolomic and Functional Genomic Analyses Reveal Varietal Differences in Bioactive Compounds of Cooked Rice
Source: PLoS One. 2010 Sep 23;5(9):e12915. doi: 10.1371/journal.pone.0012915 (PMC2944836; doi:10.1371/journal.pone.0012915)
Supplement: Table S1 — Genes associated with linolenic acid, phenolics, phytosterol, and vitamin E synthesis. (0.18 MB PDF) [file pone.0012915.s002.pdf]

Table S1. Genes associated with linolenic acid, phenolics, phytosterol, and vitamin E synthesis.

LocusID	Enzymatic activity	SNPID	Class	Pathway
LOC_Os07g49310	omega-3 fatty acid desaturase	TBGI337504	Linolenic acid	lipid desaturation
LOC_Os07g49310	omega-3 fatty acid desaturase	TBGI337502	Linolenic acid	lipid desaturation
LOC_Os08g34220	omega-6 fatty acid desaturase	TBGI358296	Linolenic acid	lipid desaturation
LOC_Os08g34220	omega-6 fatty acid desaturase	TBGI358297	Linolenic acid	lipid desaturation
LOC_Os08g34220	omega-6 fatty acid desaturase	TBGI358298	Linolenic acid	lipid desaturation
LOC_Os08g34220	omega-6 fatty acid desaturase	TBGI358299	Linolenic acid	lipid desaturation
LOC_Os08g34220	omega-6 fatty acid desaturase	TBGI358300	Linolenic acid	lipid desaturation
LOC_Os08g34220	omega-6 fatty acid desaturase	TBGI358301	Linolenic acid	lipid desaturation
LOC_Os02g48560	omega-6 fatty acid desaturase, endoplasmic reticulum isozyme 2, putative, expressed	no SNP	Linolenic acid	lipid desaturation
LOC_Os03g18070	omega-3 fatty acid desaturase	no SNP	Linolenic acid	lipid desaturation
LOC_Os07g23410	omega-6 fatty acid desaturase, endoplasmic reticulum isozyme 2, putative, expressed	no SNP	Linolenic acid	lipid desaturation
LOC_Os07g23430	omega-6 fatty acid desaturase, endoplasmic reticulum isozyme 2, putative, expressed	no SNP	Linolenic acid	lipid desaturation
LOC_Os11g01340	omega-3 fatty acid desaturase	no SNP	Linolenic acid	lipid desaturation
LOC_Os12g01370	omega-3 fatty acid desaturase	no SNP	Linolenic acid	lipid desaturation
LOC_Os07g49310	omega-3 fatty acid desaturase	TBGI337504	Linolenic acid	lipid desaturation
LOC_Os07g49310	omega-3 fatty acid desaturase	TBGI337502	Linolenic acid	lipid desaturation
LOC_Os01g60450	trans-cinnamate 4-monooxygenase	TBGI059140	Phenolic	phenylpropanoid biosynthesis
LOC_Os02g41630	phenylalanine ammonia-lyase	TBGI107353	Phenolic	phenylpropanoid biosynthesis
LOC_Os02g41670	phenylalanine ammonia-lyase	TBGI107368	Phenolic	phenylpropanoid biosynthesis
LOC_Os02g41680	phenylalanine ammonia-lyase	TBGI107403	Phenolic	phenylpropanoid biosynthesis
LOC_Os02g46970	4-coumarate-CoA ligase	TBGI111976	Phenolic	phenylpropanoid biosynthesis
LOC_Os03g02180	ferulate 5-hydroxylase	TBGI124364	Phenolic	phenylpropanoid biosynthesis
LOC_Os06g24180	ferulate 5-hydroxylase	TBGI289865	Phenolic	phenylpropanoid biosynthesis
LOC_Os06g24180	ferulate 5-hydroxylase	TBGI289869	Phenolic	phenylpropanoid biosynthesis
LOC_Os08g14760	4-coumarate-CoA ligase	TBGI347005	Phenolic	phenylpropanoid biosynthesis
LOC_Os12g33610	phenylalanine ammonia-lyase	TBGI479943	Phenolic	phenylpropanoid biosynthesis
LOC_Os12g33610	phenylalanine ammonia-lyase	TBGI479945	Phenolic	phenylpropanoid biosynthesis
LOC_Os02g41630	phenylalanine ammonia-lyase	TBGI107339	Phenolic	phenylpropanoid biosynthesis
LOC_Os02g41630	phenylalanine ammonia-lyase	TBGI107343	Phenolic	phenylpropanoid biosynthesis
LOC_Os02g41630	phenylalanine ammonia-lyase	TBGI107344	Phenolic	phenylpropanoid biosynthesis
LOC_Os02g41630	phenylalanine ammonia-lyase	TBGI107349	Phenolic	phenylpropanoid biosynthesis
LOC_Os02g41630	phenylalanine ammonia-lyase	TBGI107350	Phenolic	phenylpropanoid biosynthesis
LOC_Os02g41630	phenylalanine ammonia-lyase	TBGI107351	Phenolic	phenylpropanoid biosynthesis
LOC_Os02g41630	phenylalanine ammonia-lyase	TBGI107352	Phenolic	phenylpropanoid biosynthesis
LOC_Os02g41670	phenylalanine ammonia-lyase	TBGI107357	Phenolic	phenylpropanoid biosynthesis
LOC_Os02g41680	phenylalanine ammonia-lyase	TBGI107397	Phenolic	phenylpropanoid biosynthesis
LOC_Os02g41680	phenylalanine ammonia-lyase	TBGI107399	Phenolic	phenylpropanoid biosynthesis
LOC_Os03g02180	ferulate 5-hydroxylase	TBGI124360	Phenolic	phenylpropanoid biosynthesis
LOC_Os02g41680	phenylalanine ammonia-lyase	TBGI107408	Phenolic	phenylpropanoid biosynthesis
LOC_Os01g60450		TBGI059143	Phenolic	phenylpropanoid biosynthesis
LOC_Os01g60450		TBGI059145	Phenolic	phenylpropanoid biosynthesis
LOC_Os02g46970	4-coumarate-CoA ligase	TBGI111975	Phenolic	phenylpropanoid biosynthesis
LOC_Os06g24180	ferulate 5-hydroxylase	TBGI289866	Phenolic	phenylpropanoid biosynthesis
LOC_Os06g24180	ferulate 5-hydroxylase	TBGI289873	Phenolic	phenylpropanoid biosynthesis
LOC_Os06g24180	ferulate 5-hydroxylase	TBGI289874	Phenolic	phenylpropanoid biosynthesis
LOC_Os06g24180	ferulate 5-hydroxylase	TBGI289875	Phenolic	phenylpropanoid biosynthesis
LOC_Os08g14760	4-coumarate-CoA ligase	TBGI347006	Phenolic	phenylpropanoid biosynthesis
LOC_Os08g14760	4-coumarate-CoA ligase	TBGI347008	Phenolic	phenylpropanoid biosynthesis
LOC_Os08g14760	4-coumarate-CoA ligase	TBGI347011	Phenolic	phenylpropanoid biosynthesis
LOC_Os01g60450	phenylpropanoid biosynthesis	TBGI059141	Phenolic	phenylpropanoid biosynthesis
LOC_Os01g60450	phenylpropanoid biosynthesis	TBGI059147	Phenolic	phenylpropanoid biosynthesis
LOC_Os01g60450.6	phenylpropanoid biosynthesis	TBGI059151	Phenolic	phenylpropanoid biosynthesis
LOC_Os02g41630	phenylalanine ammonia-lyase	TBGI107354	Phenolic	phenylpropanoid biosynthesis
LOC_Os02g41630	phenylalanine ammonia-lyase	TBGI107355	Phenolic	phenylpropanoid biosynthesis
LOC_Os02g41670	phenylalanine ammonia-lyase	TBGI107371	Phenolic	phenylpropanoid biosynthesis
LOC_Os02g41680	phenylalanine ammonia-lyase	TBGI107406	Phenolic	phenylpropanoid biosynthesis
LOC_Os05g35290	phenylalanine ammonia-lyase	TBGI250746	Phenolic	phenylpropanoid biosynthesis
LOC_Os05g35290	phenylalanine ammonia-lyase	TBGI250747	Phenolic	phenylpropanoid biosynthesis
LOC_Os06g24180	ferulate 5-hydroxylase	TBGI289867	Phenolic	phenylpropanoid biosynthesis
LOC_Os06g24180	ferulate 5-hydroxylase	TBGI289868	Phenolic	phenylpropanoid biosynthesis

LOC_Os06g24180	ferulate 5-hydroxylase	TBGI289872	Phenolic	phenylpropanoid biosynthesis
LOC_Os08g14760	4-coumarate-CoA ligase	TBGI347003	Phenolic	phenylpropanoid biosynthesis
LOC_Os08g14760	4-coumarate-CoA ligase	TBGI347004	Phenolic	phenylpropanoid biosynthesis
LOC_Os08g14760	4-coumarate-CoA ligase	TBGI347009	Phenolic	phenylpropanoid biosynthesis
LOC_Os08g14760	4-coumarate-CoA ligase	TBGI347010	Phenolic	phenylpropanoid biosynthesis
LOC_Os12g33610	phenylalanine ammonia-lyase	TBGI479929	Phenolic	phenylpropanoid biosynthesis
LOC_Os12g33610	phenylalanine ammonia-lyase	TBGI479935	Phenolic	phenylpropanoid biosynthesis
LOC_Os12g33610	phenylalanine ammonia-lyase	TBGI479941	Phenolic	phenylpropanoid biosynthesis
LOC_Os12g33610	phenylalanine ammonia-lyase	TBGI479944	Phenolic	phenylpropanoid biosynthesis
LOC_Os12g33610	phenylalanine ammonia-lyase	TBGI479949	Phenolic	phenylpropanoid biosynthesis
LOC_Os04g43760	phenylalanine ammonia-lyase	no SNP	Phenolic	phenylpropanoid biosynthesis
LOC_Os06g06980	caffeoyl-CoA O-methyltransferase	no SNP	Phenolic	phenylpropanoid biosynthesis
LOC_Os08g38900	caffeoyl-CoA O-methyltransferase	no SNP	Phenolic	phenylpropanoid biosynthesis
LOC_Os08g38910	caffeoyl-CoA O-methyltransferase	no SNP	Phenolic	phenylpropanoid biosynthesis
LOC_Os08g38920	caffeoyl-CoA O-methyltransferase	no SNP	Phenolic	phenylpropanoid biosynthesis
LOC_Os09g30360	caffeoyl-CoA O-methyltransferase	no SNP	Phenolic	phenylpropanoid biosynthesis
LOC_Os10g36848	ferulate 5-hydroxylase	no SNP	Phenolic	phenylpropanoid biosynthesis
LOC_Os11g48110	phenylalanine ammonia-lyase	no SNP	Phenolic	phenylpropanoid biosynthesis
LOC_Os06g24180	ferulate 5-hydroxylase	TBGI289864	Phenolic	phenylpropanoid biosynthesis
LOC_Os01g27490	leucoanthocyanidin dioxygenase	TBGI028393	Phenolic	flavanoid biosynthesis
LOC_Os01g27490	leucoanthocyanidin dioxygenase	TBGI028394	Phenolic	flavanoid biosynthesis
LOC_Os01g27490	leucoanthocyanidin dioxygenase	TBGI028395	Phenolic	flavanoid biosynthesis
LOC_Os01g27490	leucoanthocyanidin dioxygenase	TBGI028396	Phenolic	flavanoid biosynthesis
LOC_Os06g42130	leucoanthocyanidin dioxygenase	TBGI304688	Phenolic	flavanoid biosynthesis
LOC_Os06g42130	leucoanthocyanidin dioxygenase	TBGI304691	Phenolic	flavanoid biosynthesis
LOC_Os01g27490	leucoanthocyanidin dioxygenase	TBGI028392	Phenolic	flavanoid biosynthesis
LOC_Os03g60509	putative chalcone isomerase	TBGI173576	Phenolic	flavanoid biosynthesis
LOC_Os01g27490	leucoanthocyanidin dioxygenase	TBGI028398	Phenolic	flavanoid biosynthesis
LOC_Os01g27490	leucoanthocyanidin dioxygenase	TBGI028400	Phenolic	flavanoid biosynthesis
LOC_Os01g44260	dihydroflavonol-4-reductase	no SNP	Phenolic	flavanoid biosynthesis
LOC_Os04g56700	naringenin,2-oxoglutarate 3-dioxygenase	no SNP	Phenolic	flavanoid biosynthesis
LOC_Os07g11440	putative chalcone synthase	no SNP	Phenolic	flavanoid biosynthesis
LOC_Os10g17260	flavonoid 3-monooxygenase	no SNP	Phenolic	flavanoid biosynthesis
LOC_Os11g32650	putative chalcone synthase	no SNP	Phenolic	flavanoid biosynthesis
LOC_Os09g19734	isochorismate synthase 1, chloroplast precursor, putative, expressed	no SNP	Phenolic	via chorismate, pyruvate pathway
LOC_Os08g35310	isoflavone-7-O-methytransferase 9	TBGI359460	Phenolic	flavanoid biosynthesis
LOC_Os08g35310	isoflavone-7-O-methytransferase 9	TBGI359464	Phenolic	flavanoid biosynthesis
LOC_Os08g35310	isoflavone-7-O-methytransferase 9	TBGI359466	Phenolic	flavanoid biosynthesis
LOC_Os08g35310	isoflavone-7-O-methytransferase 9	TBGI359467	Phenolic	flavanoid biosynthesis
LOC_Os10g16974	flavonoid 3-hydroxylase	TBGI400908	Phenolic	leucodelphinidin biosynthesis (dihydrotricetin (demthylated tricin))
LOC_Os10g16974	flavonoid 3-hydroxylase	TBGI400906	Phenolic	leucodelphinidin biosynthesis (dihydrotricetin (demthylated tricin))
LOC_Os08g35310	isoflavone-7-O-methytransferase 9	TBGI359462	Phenolic	flavanoid biosynthesis
LOC_Os08g35310	isoflavone-7-O-methytransferase 9	TBGI359463	Phenolic	flavanoid biosynthesis
LOC_Os10g16974	flavonoid 3-hydroxylase	TBGI400913	Phenolic	leucodelphinidin biosynthesis (dihydrotricetin (demthylated tricin))
LOC_Os10g16974	flavonoid 3-hydroxylase	TBGI400915	Phenolic	leucodelphinidin biosynthesis (dihydrotricetin (demthylated tricin))
LOC_Os10g16974	flavonoid 3-hydroxylase	TBGI400916	Phenolic	leucodelphinidin biosynthesis (dihydrotricetin (demthylated tricin))
LOC_Os10g16974	flavonoid 3-hydroxylase	TBGI400920	Phenolic	leucodelphinidin biosynthesis (dihydrotricetin (demthylated tricin))
LOC_Os08g35310	isoflavone-7-O-methytransferase 9	TBGI359465	Phenolic	flavanoid biosynthesis
LOC_Os09g09230	dihydroflavonol-4-reductase, putative	no SNP	Phenolic	leucodelphinidin biosynthesis (dihydrotricetin (demthylated tricin))
LOC_Os10g17260	flavonoid 3-hydroxylase	no SNP	Phenolic	leucodelphinidin biosynthesis (dihydrotricetin (demthylated tricin))
LOC_Os05g34380	cytochrome P450 51, putative	TBGI249857	Phytosterol	sterol synthesis

LOC_Os07g37980	cytochrome P450 51, putative	TBGI329832	Phytosterol	sterol synthesis
LOC_Os03g59040	farnesyl-diphosphate farnesyltransferase	TBGI172275	Phytosterol	sterol synthesis
LOC_Os03g59040	farnesyl-diphosphate farnesyltransferase	TBGI172276	Phytosterol	sterol synthesis
LOC_Os03g59040	farnesyl-diphosphate farnesyltransferase	TBGI172274	Phytosterol	sterol synthesis
LOC_Os07g10130	farnesyl-diphosphate farnesyltransferase	TBGI317153	Phytosterol	sterol synthesis
LOC_Os07g10600	sterol 24-C-methyltransferase	TBGI317459	Phytosterol	sterol synthesis
LOC_Os07g10600	sterol 24-C-methyltransferase	TBGI317461	Phytosterol	sterol synthesis
LOC_Os07g37970	cytochrome P450 51, putative	TBGI329813	Phytosterol	sterol synthesis
LOC_Os07g37980	cytochrome P450 51, putative	TBGI329833	Phytosterol	sterol synthesis
LOC_Os07g37980	cytochrome P450 51, putative	TBGI329834	Phytosterol	sterol synthesis
LOC_Os07g37980	cytochrome P450 51, putative	TBGI329836	Phytosterol	sterol synthesis
LOC_Os07g37980	cytochrome P450 51, putative	TBGI329837	Phytosterol	sterol synthesis
LOC_Os07g37980	cytochrome P450 51, putative	TBGI329840	Phytosterol	sterol synthesis
LOC_Os07g37980	cytochrome P450 51, putative	TBGI329843	Phytosterol	sterol synthesis
LOC_Os09g39220	C-14 sterol reductase	TBGI391322	Phytosterol	sterol synthesis
LOC_Os09g39220	C-14 sterol reductase	TBGI391323	Phytosterol	sterol synthesis
LOC_Os09g39220	C-14 sterol reductase	TBGI391329	Phytosterol	sterol synthesis
LOC_Os05g34380	cytochrome P450 51, putative	TBGI249853	Phytosterol	sterol synthesis
LOC_Os05g34380	cytochrome P450 51, putative	TBGI249858	Phytosterol	sterol synthesis
LOC_Os05g34380	cytochrome P450 51, putative	TBGI249862	Phytosterol	sterol synthesis
LOC_Os07g37970	cytochrome P450 51, putative	TBGI329817	Phytosterol	sterol synthesis
LOC_Os01g01369	3-beta-hydroxysteroid-delta-isomerase, putative, expressed	no SNP	Phytosterol	sterol synthesis
LOC_Os01g25189	C-14 sterol reductase	no SNP	Phytosterol	sterol synthesis
LOC_Os02g04760	cycloartenol synthase, putative	no SNP	Phytosterol	sterol synthesis
LOC_Os02g26650	sterol delta7 reductase	no SNP	Phytosterol	sterol synthesis
LOC_Os03g04340	S-adenosylmethionine-dependent methyltransferase	no SNP	Phytosterol	sterol synthesis
LOC_Os05g14800	cycloartenol synthase, putative	no SNP	Phytosterol	sterol synthesis
LOC_Os07g28110	cytochrome P450 51, putative	no SNP	Phytosterol	sterol synthesis
LOC_Os07g28160	cytochrome P450 51, putative	no SNP	Phytosterol	sterol synthesis
LOC_Os11g18310	cycloartenol synthase, putative	no SNP	Phytosterol	sterol synthesis
LOC_Os11g18340	cycloartenol synthase, putative	no SNP	Phytosterol	sterol synthesis
LOC_Os11g19700	cycloeucalenol cycloisomerase	no SNP	Phytosterol	sterol synthesis
LOC_Os02g17920	4-hydroxyphenylpyruvate dioxygenase	TBGI090438	Vitamin E	vitamin E biosynthesis
LOC_Os02g17920	4-hydroxyphenylpyruvate dioxygenase	TBGI090439	Vitamin E	vitamin E biosynthesis
LOC_Os02g17920	4-hydroxyphenylpyruvate dioxygenase	TBGI090428	Vitamin E	vitamin E biosynthesis
LOC_Os02g17920	4-hydroxyphenylpyruvate dioxygenase	TBGI090429	Vitamin E	vitamin E biosynthesis
LOC_Os02g17920	4-hydroxyphenylpyruvate dioxygenase	TBGI090441	Vitamin E	vitamin E biosynthesis
LOC_Os08g09250	4-hydroxyphenylpyruvate dioxygenase	TBGI343503	Vitamin E	vitamin E biosynthesis
LOC_Os08g09250	4-hydroxyphenylpyruvate dioxygenase	TBGI343504	Vitamin E	vitamin E biosynthesis
LOC_Os02g07160	4-hydroxyphenylpyruvate dioxygenase	TBGI081638	Vitamin E	vitamin E biosynthesis
LOC_Os02g17650	tocopherol cyclase	TBGI090186	Vitamin E	vitamin E biosynthesis
LOC_Os02g17650	tocopherol cyclase	TBGI090187	Vitamin E	vitamin E biosynthesis
LOC_Os02g17650	tocopherol cyclase	TBGI090190	Vitamin E	vitamin E biosynthesis
LOC_Os02g17650	tocopherol cyclase	TBGI090191	Vitamin E	vitamin E biosynthesis
LOC_Os02g17650	tocopherol cyclase	TBGI090193	Vitamin E	vitamin E biosynthesis
LOC_Os02g17650	tocopherol cyclase	TBGI090195	Vitamin E	vitamin E biosynthesis
LOC_Os02g17920	4-hydroxyphenylpyruvate dioxygenase	TBGI090432	Vitamin E	vitamin E biosynthesis
LOC_Os02g17920	4-hydroxyphenylpyruvate dioxygenase	TBGI090436	Vitamin E	vitamin E biosynthesis
LOC_Os02g17920	4-hydroxyphenylpyruvate dioxygenase	TBGI090437	Vitamin E	vitamin E biosynthesis
LOC_Os05g14194	4-hydroxyphenylpyruvate dioxygenase	TBGI236102	Vitamin E	vitamin E biosynthesis
LOC_Os05g14194	4-hydroxyphenylpyruvate dioxygenase	TBGI236103	Vitamin E	vitamin E biosynthesis

LOC_Os05g14194	4-hydroxyphenylpyruvate dioxygenase	TBGI236108	Vitamin E	vitamin E biosynthesis
LOC_Os05g14194	4-hydroxyphenylpyruvate dioxygenase	TBGI236109	Vitamin E	vitamin E biosynthesis
LOC_Os02g17650	tocopherol cyclase	TBGI090196	Vitamin E	vitamin E biosynthesis
LOC_Os12g42090	tocopherol cyclase	TBGI489786	Vitamin E	vitamin E biosynthesis
LOC_Os02g47310	tocopherol O-methyltransferase	no SNP	Vitamin E	vitamin E Biosynthesis
LOC_Os06g43880	homogentisic acid geranylgeranyl transferase	no SNP	Vitamin E	vitamin E Biosynthesis
LOC_Os06g44840	homogentisate phytyltransferase	no SNP	Vitamin E	vitamin E biosynthesis
LOC_Os07g49310	omega-3 fatty acid desaturase	TBGI337504	Linolenic acid	glycolipid desaturation
LOC_Os07g49310	omega-3 fatty acid desaturase	TBGI337502	Linolenic acid	glycolipid desaturation
